# Age-Related Macular Degeneration and Smoking Cessation Advice by Eye Care Providers: A Pilot Study

**Published:** 2011-10-15

**Authors:** Alberto J. Caban-Martinez, Evelyn P. Davila, Kathryn E. McCollister, Lora E. Fleming, Diane D. Zheng, Byron L. Lam, Sander R. Dubovy, David J. Lee

**Affiliations:** Department of Epidemiology and Public Health, University of Miami, Miller School of Medicine, Clinical Research Building; Department of Epidemiology and Public Health, University of Miami, Miller School of Medicine, Miami, Florida; Department of Epidemiology and Public Health, University of Miami, Miller School of Medicine, Miami, Florida; Department of Epidemiology and Public Health, University of Miami, Miller School of Medicine, Miami, Florida; Department of Epidemiology and Public Health, University of Miami, Miller School of Medicine, Miami, Florida; Department of Ophthalmology, University of Miami, Miller School of Medicine, Miami, Florida; Department of Ophthalmology, University of Miami, Miller School of Medicine, Miami, Florida; Department of Epidemiology and Public Health and Department of Ophthalmology, University of Miami, Miller School of Medicine, Miami, Florida

## Abstract

Smoking is a modifiable risk factor for age-related macular degeneration (AMD), the leading cause of irreversible vision loss in the United States. We conducted a pilot study among eye care providers and AMD patients to assess smoking cessation preferences and cessation services offered at a large academic medical center. Most patients who smoke reported never being advised to quit smoking, although most eye care providers reported that they had advised smokers to quit. Two-thirds of providers expressed a desire for additional training and resources to support patient quit attempts, indicating the need for the integration of smoking cessation opportunities in the clinic setting.

## Objective

In the United States, age-related macular degeneration (AMD) is the leading cause of severe and irreversible vision loss, affecting more than 9.1 million people. Because of the increasing number of older people in the US population, this number is expected to increase to 17.8 million by 2050 (1). Treatment options for "dry" AMD (eg, loss of retinal pigment and photoreceptors in the central part of the eye) are limited; therefore, addressing the few modifiable risk factors of AMD such as smoking is important (2). Little is known about the level of smoking cessation services offered to patients who smoke and who are being treated for AMD and the smoking cessation preferences of these patients (2). The purpose of this pilot study was to assess tobacco use and smoking cessation preferences of AMD patients and level and preference of smoking cessation services offered by their eye care providers at a large academic medical center.

## Methods

The institutional review board at the University of Miami approved this pilot study. In June 2009, we sent clinical faculty, fellows, and residents at the Bascom Palmer Eye Institute (BPEI) an e-mail message (and 1 follow-up reminder), extending an invitation to participate in a brief, anonymous Web-based, 16-item survey (SurveyMonkey, Palo Alto, California). We used a modified question set obtained from the Association of American Medical Colleges' national smoking cessation survey of primary health care providers (3). The survey inquired about awareness and clinical practice of treating patients who smoke, specific eye care provider medical training, and provider sociodemographic characteristics.

During the same time, study team interviewers approached AMD patients who were visiting a BPEI retinal clinic in the patient waiting area to explain study objectives, assess interest in participating, and obtain verbal consent. Inclusionary participant criteria included having a diagnosis of AMD, being aged 18 years or older, and being fluent in English or Spanish. Interviewers administered an anonymous, language-sensitive (English or Spanish), 43-item paper questionnaire developed on the basis of standard tobacco use questions from the Centers for Disease Control and Prevention's Behavioral Risk Factor Surveillance System survey and the Tobacco Use Supplement of the National Cancer Institute's Current Population Survey, as well as selected study-specific vision health and eye disease questions (4,5). No participant incentives were provided. Analyses were performed using Predictive Analytics Software (SPSS, Inc, Chicago, Illinois) version 18.0.

## Results

### Eye care provider responses

The response rate for the eye care provider questionnaire was 51% (46 of 90). Forty-six percent of providers were women, 65% were faculty members, 17% were fellows, and 17% were residents; the most common age group was 30 to 39 years. Most eye care providers indicated that they seldom or periodically asked about their patients' smoking status, assessed their willingness to quit, and advised them to quit smoking (Table 1). The proportion of providers who indicated that they always engaged in these activities ranged from 7% to 28%. Eye care providers were aware that individual counseling for smoking cessation was available at the institution (94%) but were less aware that group counseling (7%) and multilingual resources were available (13%) (Table 2). Most eye care providers indicated that they would recommend these services if readily available in the clinic.

When prompted to identify which smoking cessation training or information the eye care provider would like to use, 46% wanted training on how to select self-help materials to give their patients and 39% wanted to learn how to provide social support to their patients as part of their cessation treatment. Most eye care providers indicated that their medical school education did not provide adequate training to effectively provide smoking cessation assistance to their patients (65%).

### Patient responses

The response rate for the AMD patient questionnaire was 100% (52 of 52). Respondents' age ranged from 52 to 97 years (mean age, 81 y). Forty-eight respondents were white and 4 were black; 22 reported Hispanic ethnicity. Of respondents, 19 had a high school diploma or less, and 51 reported some type of health care insurance coverage. More than half of patients (54%) were not certain whether smoking causes macular degeneration or severe visual impairment, and 17% were smokers who, on average, visited an ophthalmologist 5 times per year. Nearly 90% of smokers reported never being advised to quit by their eye care provider, although two-thirds (n = 6) reported that they were seriously considering quitting smoking in the next 6 months.

## Discussion

Epidemiologic evidence strongly suggests a causal relationship between smoking and the development of AMD (6). We found that a large proportion of AMD clinic patients were unaware of the link between smoking and eye disease. Eye care providers were interested in providing more smoking cessation services for their patients who smoke but generally reported lacking appropriate training, which indicates that opportunities exist for enhancing eye care and medical training curricula. Emerging evidence indicates that the risk of tobacco use–related eye diseases decreases substantially after smoking cessation, as suggested by the Rotterdam Study and from other scientific review articles (7-9). Therefore, enhancement of smoking cessation efforts among AMD patients should be considered an ocular-health priority, although the provision of such services should be offered to all clinic patients, given the overall deleterious health effects of continued smoking (10).

The sample size of this pilot study was small; administration of the eye care provider and patient questionnaires across a more representative sample of private practice and tertiary eye care clinics may provide additional insight into factors that influence the awareness and attitudes of patients and eye care providers in smoking cessation efforts. Finally, eye care provider survey response rates were slightly lower than those observed in national surveys of health care providers (11).

In conclusion, this study documented that both clinicians and their patients with AMD who smoke expressed a desire to facilitate and initiate quit attempts. Addiction to tobacco is increasingly considered to be a chronic condition often characterized by repeated attempts to maintain long-term abstinence (1,2). System-based approaches in which comprehensive smoking cessation services (eg, telephone quitlines) are embedded in integrated patient-care models have shown promising results (3), yet such a comprehensive program has yet to be established and validated in an ocular clinic setting. Development of such a program in the context of the setting of busy eye care providers will not only help create awareness of the relationship between smoking and ocular disease risk but may also reduce the overall burden of tobacco use–associated disease in this high-risk population.

## Figures and Tables

**Table 1:** Prevalence of Eye Care Providers' (n = 46) Smoking Status Assessment, Cessation Advice, and Treatment Practices, Florida, 2009

Smoking Intervention Question	Reported Frequency of Activity		

	Always, n^a^ (%)	Periodically/Seldom, n^a^ (%)	Never, n^a^ (%)
Ask about smoking status	6 (13)	37 (80)	3 (7)
Assess patient willingness to quit smoking	3 (7)	35 (76)	8 (17)
Advise patients to stop smoking	13 (28)	30 (65)	3 (7)
Refer patients who smoke to other providers for cessation	0	27 (59)	19 (41)
Recommend nicotine replacement therapy	0	23 (50)	23 (50)
Prescribe other medication	0	3 (7)	43 (94)
Provide brochures/self-help materials	0	3 (7)	43 (94)
Arrange follow-up visits with patient to address smoking	0	7 (15)	39 (85)
Monitor patient progress in attempting to quit	3 (7)	16 (35)	27 (59)

a Differences in subtotal population sample due to item nonresponse or missing.

**Table 2.** Providers' Awareness of Smoking Cessation Resources Available at Their Institution, Florida, 2009

**Smoking Cessation Resource**	**Available in Clinic, n** a **(%)**	Would Use if Available, n^a^ **(%)**
Individual counseling	43 (94)	43 (94)
Group programs	3 (7)	28 (61)
Multilingual resources	6 (13)	29 (63)
Web-based smoking cessation programs	4 (9)	29 (63)

a Differences in subtotal population sample due to item nonresponse or missing.
